# Comparative evaluation of patients’ and physicians’ satisfaction with interferon beta-1b therapy

**DOI:** 10.1186/s12883-016-0705-1

**Published:** 2016-09-21

**Authors:** Uwe Klaus Zettl, Ulrike Bauer-Steinhusen, Thomas Glaser, Klaus Hechenbichler, Michael Hecker, for the Study Group

**Affiliations:** 1 Department of Neurology, Neuroimmunology Section, University of Rostock, Gehlsheimer Str. 20, 18147 Rostock, Germany; 2 Neurology, Immunology, and Ophthalmology, Bayer Vital GmbH, Leverkusen, Germany; 3 Institute Dr. Schauerte, Munich, Germany

**Keywords:** Multiple Sclerosis, Interferon beta-1b, Treatment Satisfaction, Patient-Physician Relationship, Therapy Adherence

## Abstract

**Background:**

Due to the preventive nature of disease-modifying therapies for multiple sclerosis, treatment success particularly depends on adherence to therapeutic regimens and patients’ perception of treatment efficacy. The latter is strongly influenced by the confidence in the involved health care professionals and the relationship to the treating physician.

**Methods:**

In this report, we considered physicians’ and patients’ evaluation of satisfaction with interferon beta-1b treatment efficacy for assessing the congruence in ratings. Data were queried in a study conducted between 2009 and 2013.

**Results:**

After 6 months of therapy, > 80 % of the patients and physicians (*N* = 445) showed high degrees of satisfaction regarding interferon beta-1b treatment, with only few physicians and patients (≤2.0 %) rating “not satisfied”. The proportion of patients rating with the same category as their physicians was similar after 6 months (47 % congruence) and at the 24 months/study end visit (49 %). Discrepancies between ratings were observed with respect to study end: for patients with premature study end, more patients and physicians rated being not satisfied with the therapy, accompanied by a considerably lower congruence of 33 % compared to 54 % for patients receiving the therapy for at least 2 years and completing the study regularly.

**Conclusions:**

Regular communication between physicians and patients about their perception of therapy might improve alignment of treatment evaluation and could result in increased therapy persistence. In addition, patients’ willingness to perform a long-term therapy − even in the absence of disease symptoms − might be promoted by repeated exchange between health care providers and patients with regard to realistic treatment expectations.

**Trial registration:**

ClinicalTrials.gov NCT00902135 (registered May 13, 2009).

**Electronic supplementary material:**

The online version of this article (doi:10.1186/s12883-016-0705-1) contains supplementary material, which is available to authorized users.

## Background

Multiple sclerosis (MS) is a chronic inflammatory disease of the central nervous system and affects more than 2.5 million individuals worldwide [1]. Due to the chronic nature of the disease and the preventive character of approved disease-modifying drugs (DMD), long-term treatment of patients is indispensable. Various therapeutic agents with diverse routes of administration and considerable differences in the safety profiles are available. Treatment with first-line DMD such as interferon beta-1b is well-established and well-tolerated [2, 3]. However, adherence to treatment regimens and persistence to therapy play central roles for therapeutic success. In particular, adverse events and (perceived) lack of efficacy often interfere with treatment initiation and long-term treatment continuation [4, 5]. Management of common adverse events − in part through specific nursing support programs and/or by dose titration at therapy onset − as well as education of patients with respect to reasonable expectations and knowledge of associated risks and benefits can prevent therapy discontinuation and increase satisfaction of patients [2, 4, 6].

Patients‘ and physicians‘ opinions on therapy might differ remarkably, as known from a survey among diabetic patients who were recommended to start long-term insulin therapy [7]. Therefore, recognizing and understanding patients‘ concerns regarding therapeutic options can be critical for therapy initiation and subsequent compliance [8]. In addition, accommodating patients‘ preferences regarding the physician’s role (e.g., the desire to be or not to be involved in the decision making, and the mode of communication) might have an impact on patient adherence to an agreed treatment as well as on health benefits resulting from sense of autonomy and satisfaction with the decision [9].

Here, we report our results from a prospective national cohort study with comparison of patients’ and physicians’ satisfaction with interferon beta-1b therapy.

## Methods

The data are based on a prospective, non-interventional, open-label, national cohort study, BETAPATH, which was designed to assess the utility of an electronic patient diary in MS patients treated with interferon beta-1b (Betaferon®). The study was conducted in Germany between 04/2009 and 12/2013 [10]. Prerequisites for enrollment were confirmed clinically isolated syndrome (CIS) or relapsing-remitting MS (RRMS) and appropriate age (≥18 years). In addition, patients were included in this study only after initiation of treatment with interferon beta-1b and completion of the initial dose escalation. Patients were followed for a period of 24 months after the baseline visit and had to give written informed consent. The study was conducted in accordance with the Declaration of Helsinki, approved by the Ethics Committee of the Ärztekammer Nordrhein (03/2009), and registered at ClinicalTrials.gov (NCT00902135).

Standardized case report forms (CRF) were used at the initial visit, all follow-up visits, and the final visit. The final visit (study end) was after 24 months for study completers, whereas for patients who discontinued the study prematurely, it was the documented end of observation at any previous time point. In the CRF, the treating physicians recorded the date, disease course and progression, cognitive state, and changes in therapy [10]. At follow-up visits, the physicians also evaluated their satisfaction concerning the efficacy of interferon beta-1b therapy with regard to the individual case. In addition, at 6, 12, and 24 months/study end, patients were queried on separate questionnaires addressing health-related issues such as quality of life, fatigue, depression, and satisfaction with therapy. Satisfaction ratings were performed by use of 4 categories (very satisfied, satisfied, neutral, or not satisfied). For the analysis of congruence in these ratings, cases with missing data were omitted, physicians’ ratings were taken as a basis, and the proportion of patients rating with the same category was calculated.

Adherence to the treatment regimen as well as side effects were investigated within the BETAPATH study as well. To assess the regularity of treatment for the individual patients, the number of injections as recorded in the electronic diary was analyzed. Non-adherence was defined as >5 missed injections per 6-month interval as previously described [11]. Drug-related adverse events (AE), which occurred during or after injection of interferon beta-1b and not later than 28 days after the end of observation, were recorded using the MedDRA coding system. Baseline characteristics and data on adherence and persistence to treatment have been published elsewhere [10, 11].

## Results

### Satisfaction with interferon beta-1b therapy

A total of 669 patients participated in the BETAPATH study [11]. For the present study, a subset of this cohort was considered, because the question on satisfaction with interferon beta-1b therapy was not in all cases answered simultaneously by both the patient and the physician. Satisfaction with the therapy was evaluated after 6 months by *N* = 445 patients (125 men and 320 women) and their physicians. At study end, satisfaction was evaluated by *N* = 353 physicians/patients (98 men and 255 women) (Table 1). The regular study end after 24 months was achieved by *N* = 266 of these 353 cases, whereas *N* = 87 patients terminated the study prematurely (Table 2, Additional file 1). Reasons for premature study end were diverse: *N* = 70 patients quit the treatment, *N* = 7 patients were tired of questionnaires, *N* = 3 patients moved to another place, and *N* = 7 indicated other reasons.

**Table 1:** Clinical and demographic characteristics of the patients at study onset

	Evaluation of satisfaction after 6 months (*N* = 445)	Evaluation of satisfaction at study end (*N* = 353)
Sex, N (%)		
Male	125 (28.1)	98 (27.8)
Female	320 (71.9)	255 (72.2)
Age (years), mean (SD)	38.5 (10.4)	39.2 (10.1)
Time since diagnosis, years (SD)	3.3 (5.8)	3.6 (6.1)
Number of relapses (last 2 years), mean (SD)	1.6 (1.2)	1.6 (1.4)
CIS, N (%)	22 (4.9)	16 (4.5)
RRMS, N (%)	423 (95.1)	337 (95.5)
EDSS, mean (SD)	2.0 (1.4)	2.0 (1.4)
Previous treatment, N (%)		
No	334 (75.1)	258 (73.1)
Yes	110 (24.7)	94 (26.6)
Missing	1 (0.2)	1 (0.3)

*CIS* clinically isolated syndrome, *EDSS* expanded disability status scale, *RRMS* relapsing-remitting multiple sclerosis, *SD* standard deviation

**Table 2:** Congruence of ratings of patients (questionnaire) and their physicians (CRF)

	6 Months			24 Months/study end			Regular study end			Premature study end		
		Congruence			Congruence			Congruence			Congruence	
	Total N	N	%	Total N	N	%	Total N	N	%	Total N	N	%
Total	445			353			266			87		
Very satisfied	140	88	62.9	125	79	63.2	112	72	64.3	13	7	53.8
Satisfied	246	107	43.5	154	73	47.4	124	65	52.4	30	8	26.7
Neutral	40	9	22.5	37	11	29.7	18	5	27.8	19	6	31.6
Not satisfied	9	3	33.3	24	8	33.3	5	0	0.0	19	8	42.1
Not specified	10	2	20.0	13	1	7.7	7	1	14.3	6	0	0.0
Total congruence rate		209	47 %		172	49 %		143	54 %		29	33 %

*CRF* case report forms, *total N* total number of patients‘ and physicians‘ assessments available for congruence analysis, *N* number of patients with ratings congruent to physicians‘ evaluation

**Fig. 1:**
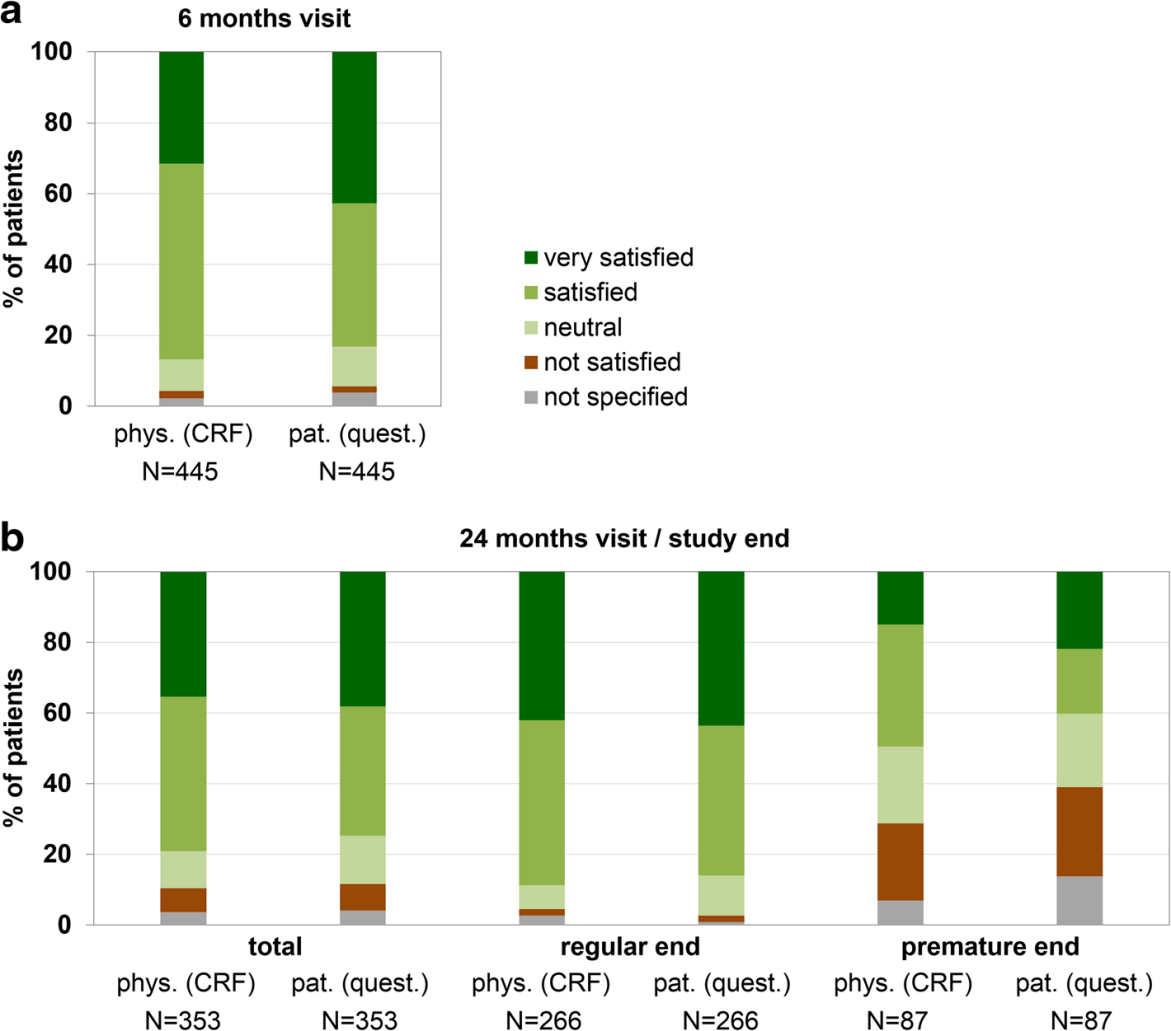
Satisfaction with interferon beta-1b therapy. **a** Physicians' and patients’ ratings of the therapy 6 months after the start of treatment as documented by the physicians in case report forms (CRF) and by the patients in a separate questionnaire. **b** Physicians' and patients’ ratings of the therapy at the final visit (24 months/study end), with substratification for patients with regular or premature study termination. pat. = patients; phys. = physicians; quest. = questionnaire

M satisfied or very satisfied with treatment efficacy throughout the study. At study end, 79.0 % of the physicians chose the ratings “very satisfied” or “satisfied”, compared to 86.7 % at the 6 months visit. Satisfaction was lower in patients’ opinion: 74.8 % of the patients rated “very satisfied” or “satisfied” according to the questionnaire at the final visit (83.1 % at the 6 months visit) (Fig. 1). Only a minority of physicians was not satisfied with the treatment (6.8 % at final visit and 2.0 % at month 6). This was similar to the patients’ point of view: 7.6 % of the patients were not satisfied at study end as documented in the questionnaires (1.8 % at the 6 months visit) (Fig. 1).

Differences in therapy evaluation were observed at the study end visit with respect to study termination: only 40.2 % of the patients who prematurely terminated the study chose the ratings “very satisfied” or “satisfied”, compared to 86.1 % of the patients receiving interferon beta-1b for at least 2 years and completing the study regularly. Differences became also obvious at study end within the category “not satisfied” (1.9 % of the patients with regular termination [*N* = 5] vs. 25.3 % of the patients with premature termination [*N* = 22]). However, absolute numbers of not satisfied patients were small. Evaluations of the physicians were similar to patients’ opinion although a higher proportion of physicians was “very satisfied/satisfied” (49.4 %) and a lower proportion rated with “not satisfied” (21.8 %) in the case that the patients terminated the study prematurely (Fig. 1b).

### Congruence of ratings

**Fig. 2:**
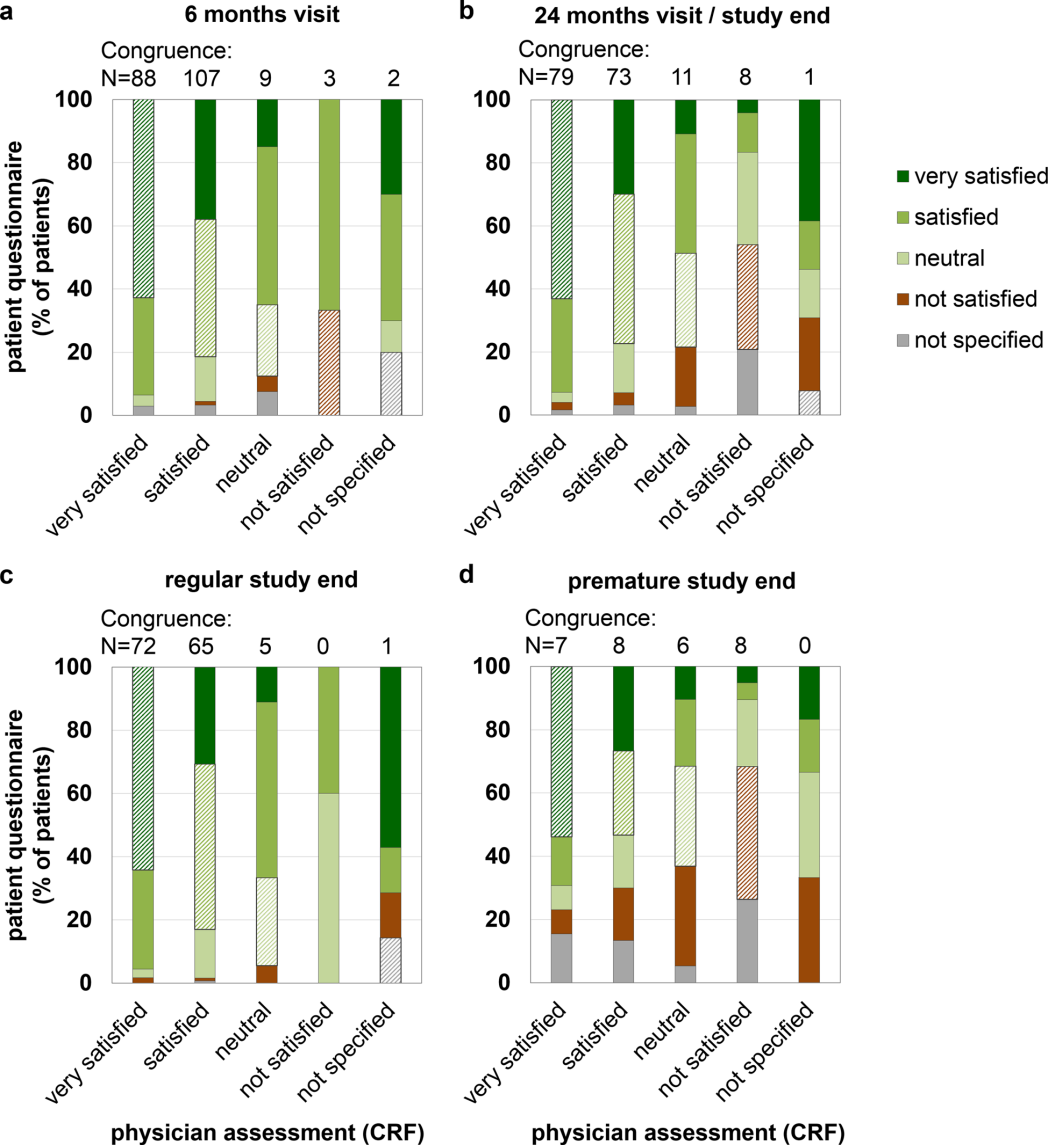
Congruence of physicians’ and patients’ evaluation of the therapy. Comparison of physicians’ and patients’ ratings of the interferon beta-1b therapy for multiple sclerosis (**a**) at the 6 months visit and (**b**) at the 24 months visit/study end, substratified for (**c**) regular and (**d**) premature study termination. CRF = case report forms; shaded areas = proportion of congruent ratings of patients and physicians

Total congruence rates (that is, the degree of agreement between patients and physicians) were 47 % after 6 months and 49 % at the 24 months/study end visit (Table 2). On closer consideration of the category “very satisfied”, the congruence between physicians’ ratings of the therapy in the CRF compared to self-documentation of the patients in the questionnaire was even higher with 62.9 % at month 6 and 63.2 % at the 24 months/study end visit. With decreasing satisfaction rating, divergences became more pronounced in the categories “neutral” and “not satisfied” (congruence rates < 35 %) (Fig. 2a, b, Table 2).

Data review with respect to study termination revealed relevant differences in congruence of therapy ratings: total congruence at study end varied from 54 % for patients with regular study end to 33 % for patients with premature study end. Patients with regular study end and their physicians concordantly chose the rating “very satisfied” in 64.3 % of cases compared to 53.8 % when considering patients with premature study end. The divergence was even greater in the categories “satisfied” (52.4 % upon regular end vs. 26.7 % upon premature end) and “not satisfied” (0 % vs. 42.1 %). In the latter case, however, the total number of patients/physicians was low (Fig. 2c, d, Table 2).

### Adherence and side effects

For the subgroup of patients who received an electronic patient diary (instead of a conventional paper diary) in the BETAPATH study, adherence to interferon beta-1b injection therapy could be evaluated. The electronic diary was used by 234 and 138 patients out of the 445 and 353 patients who rated their satisfaction with therapy (together with their physicians) after 6 months and at the final study visit, respectively (Additional file 1). Within the first 6 months, 158 patients (67.5 %) documented less than 6 missed injections, and within the last 12 months of the study, 64 patients (46.4 %) were considered adherent to the injection schedule. However, (non-)adherence was not associated with satisfaction with therapy. The percentage of patients rating “very satisfied” was > 43 % in the group of adherent patients and in the group of non-adherent patients after 6 months and after 24 months/at study end. Conversely, only < 7 % rated “not satisfied” in both patient groups and at both time points.

Drug-related AE were reported by the physicians during the observational period for 65 (18.4 %) out of the 353 patients (Additional file 1). The most common AE were injection site reactions (*N* = 24), influenza-like symptoms (*N* = 16), and headache (*N* = 6). Of the patients with AE, 16.9 % rated “very satisfied” and 15.4 % rated “not satisfied” with the therapy after 24 months/at study end. For comparison, 43.1 % and 5.9 % of the patients without AE were “very satisfied” and “not satisfied”, respectively.

## Discussion

Patients often complain about a lack of information and difficulties in making a qualified therapeutic choice. Although the treating neurologist is crucially responsible for starting or changing a DMD treatment in MS, stopping a treatment is mainly based on patients‘ decision [5]. Therefore, communication between patient and physician and close cooperation are important for a consensual opinion on a given therapy, as this has an impact on adherence and persistence to therapeutic regimens [12–14]. We investigated the patient-physician relationship by evaluating physicians’ and patients’ individual ratings of the Betaferon® therapy as well as their congruence.

The degrees of satisfaction were high: most physicians and patients (around 80 %) chose the categories “very satisfied“ or “satisfied“. More patients chose the rating “very satisfied” than physicians after 6 months. We can only speculate if this difference is due to a poor relationship between some physicians and patients or if patients feel committed to more positive opinions regarding therapy in the setting of a study. Therapy ratings of physicians and MS patients have not been addressed in parallel previously in the literature. However, in two cross-sectional surveys with respect to satisfaction with botulinum toxin for treating post-stroke spasticity, similarly high satisfaction rates were observed (88.6 % of patients and 94.2 % of physicians were satisfied with the treatment) [15].

In this study, discrepancies in patient ratings were observed after substratification for study termination. For instance, 1.9 % of the patients with regular study end were not satisfied with the therapy after 24 months, whereas, as expected, this proportion was remarkably higher among patients who discontinued the study prematurely (25.3 %). This trend was also observed in the physicians’ ratings: for patients with premature study end, 21.8 % of the respective physicians were “not satisfied”. However, after 6 months, only few patients and physicians chose this category, and no predictions with regard to treatment discontinuation of unsatisfied patients in the long-term course of the study could be made. Data from an observational study with RRMS patients indicated a significant association of adherence and overall patient satisfaction with therapy [16]. Thus, poor satisfaction with treatment might be a predictor of therapy adherence and continuation. In our data, adherence to therapy as recorded in the electronic patient diary was not related to better satisfaction ratings. This suggests that patients are not necessarily not satisfied with the treatment if they simply forget injections or if they are tired of documentation.

Patients’ and physicians’ consensus in the ratings of therapy was 47 % after 6 months and 49 % at the 24 months/study end visit. However, the total congruence rate was only 33 % with respect to patients who discontinued the study, while it was 54 % for patients completing the study and continuing the treatment with interferon beta-1b to the 24 months visit. Deficiencies in documentation (e.g., CRF documentation with temporal distance to the patient’s visit) might have influenced this outcome. Mohr and coworkers found that overoptimistic attitudes towards therapy before treatment initiation are conversely associated with therapy continuation [17]. Empathic relations between physicians and patients might reveal unrealistic opinions and help counteracting therapy discontinuation by proper education of patients. An improved adherence was observed if the treating neurologists involved the patients in the treatment plans at the onset of therapy [16] − pointing to the importance of the patient-physician relationship and shared decision making. Due to the association between patients’ perception of their relationship with physicians and patients’ satisfaction with the treatment, the involvement of MS patients is crucial for the best long-term treatment outcomes [18].

We observed that patients who were not satisfied oftentimes showed therapeutic side effects, as reflected by the higher rate of AE in this group. It is thus important to continuously educate the patients in how to prevent and manage drug-related AE [19], e.g., by rotating injection sites. In addition, other factors deteriorating patient satisfaction and treatment adherence might arise in the course of therapy, e.g., depression or changes in the personal situation [12, 13], and their impact seems to be influenced by the offered support of health care professionals [20].

The clinical practice environment is evolving, altering the daily tasks performed by physicians and other health care workers [21]. Primary care physicians spend more time supervising assistants and less time listening to patient concerns. On the contrary, the roles of nurses and medical assistants are increasingly important [22]. As physicians often cannot invest the required amount of time in the practice setting [14], MS nurses can provide personal services, and they are the preferred health care workers for a majority of patients to discuss their problems [4, 12, 23]. Besides provision of information and reinforcement of motivation to continue DMD therapy, they can also support patients in the use of autoinjectors in order to minimize anxiety associated with self-administered injections, which might be a barrier to maintenance of treatment [12]. More regular contact by telephone from health care providers may also help to confirm adequate compliance. In addition, the involvement of family members into treatment plans can improve adherence to therapy [16]. Multidisciplinary approaches in therapy and psychological support are important factors in patients’ opinion [24]. In consequence, the interpersonal relationship between the patient and the physician is one determinant of patient satisfaction among others [25, 26].

## Conclusions

In summary, overall physicians’ and patients’ satisfaction with efficacy of interferon beta-1b treatment was high. Performing a congruence analysis revealed discrepancies, especially in case of patients who later discontinued the therapy. It seems advisable to regularly assess and align patients’ and physicians’ evaluation of treatment satisfaction during scheduled appointments, with the aim of identifying reasons for dissatisfaction. Therapeutic side effects and emerging psychological symptoms (e.g., depression) might explain deviations from the physician’s opinion and provoke non-adherence to therapy [12, 19, 27]. Other health care professionals also have an impact on patients’ satisfaction with treatment. Consequently, optimized communication across multiple disciplines and coordination of continuity in medical care are needed to maintain efficacy of long-term DMD treatment in MS.

## Additional file

Additional file 1: Full data set used for congruence analysis. Satisfaction with interferon beta-1b therapy as evaluated by patients and their physicians after 6 months of treatment and at the 24 months/study end visit. For a subgroup of patients, records of an electronic patient diary allowed to assess adherence to the injection schedule in the first 6 months and in the last 12 months of the study. Moreover, the table provides the information, for which patients drug-related adverse events were reported. (XLSX 30 kb)

## Abbreviations

CIS: Clinically isolated syndrome
CRF: Case report form
DMD: Disease-modifying drug
MS: Multiple sclerosis
pat.: Patients
phys: Physicians
quest: Questionnaire
RRMS: Relapsing-remitting multiple sclerosis
